# Use of Doehlert Matrix as a Tool for High-Throughput Screening of Organic Acids and Essential Oils on Miniaturized Pork Loins, Followed by Lab-Scale Validation That Confirmed Tested Compounds Do Not Show Synergistic Effects against *Salmonella* Typhimurium

**DOI:** 10.3390/foods12214034

**Published:** 2023-11-05

**Authors:** Cristina Resendiz-Moctezuma, Arianna P. L. Fonville, Bailey N. Harsh, Matthew J. Stasiewicz, Michael J. Miller

**Affiliations:** 1 Food Science and Human Nutrition Department, University of Illinois at Urbana-Champaign, 1302 W. Pennsylvania Ave., Urbana, IL 61801, USA; cr638@cornell.edu (C.R.-M.); mstasie@illinois.edu (M.J.S.); 2 Animal Sciences Department, University of Illinois at Urbana-Champaign, 1503 Maryland Dr., Urbana, IL 61801, USA; bharsh2@illinois.edu

**Keywords:** *Salmonella*, pork, antimicrobials, essential oils, Response Surface Methodology, Design of Experiment, Doehlert matrix

## Abstract

The many possible treatments and continuously changing consumer trends present a challenge when selecting antimicrobial interventions during pork processing. Thirty-five potential antimicrobials were screened at commercial working concentrations by individually adding them to miniaturized (69 cm^3^) disks of pork loin ends, followed by inoculation with *Salmonella* Typhimurium ATCC 19585. Two organic acids and nine essential oils significantly inhibited *Salmonella* counts on pork (*p* < 0.05). However, six compounds that represent different levels of significance (*p* < 0.05–*p* < 0.0001) were selected as independent variables to build a Response Surface Methodology model based on a Doehlert matrix (Doehlert Matrix—RSM): lactic acid 1.25%, formic acid 0.25%, cumin 0.25%, clove 0.25%, peppermint 0.5%, and spearmint 0.5%. The goal of the Doehlert Matrix—RSM was to study single and paired effects of these antimicrobials on the change in *Salmonella* over 24 h. The Doehlert Matrix—RSM model predicted that lactic acid, formic acid, cumin, peppermint, and spearmint significantly reduced *Salmonella* when added alone, while no significant interactions between these antimicrobials were found. A laboratory-scale validation was carried out on pork loin end slices, which confirmed the results predicted by the model. While this screening did not identify novel synergistic combinations, our approach to screening a variety of chemical compounds by implementing a miniaturized pork loin disk model allowed us to identify the most promising antimicrobial candidates to then formally design experiments to study potential interactions with other antimicrobials.

## 1. Introduction

*Salmonella* remains a relevant foodborne pathogen for the pork industry [1]. During 2020, the European Union reported that pork and pork products were the second most commonly encountered food vehicles in confirmed salmonellosis outbreaks [2]. Therefore, pork carcass washes are currently one of the most used practices in the industry to prevent *Salmonella* Typhimurium contamination on pork meat. Currently, hot water and organic acids are the most commonly used agents to spray carcasses and cuts of pork meat. However, according to Phase II of the “Raw Pork Products Exploratory Sampling Program” by the FSIS, 8.1% of the 894 samples and 25.3% of the 1088 samples tested positive for *Salmonella* spp. on intact cuts and comminuted product, respectively [3]. Additionally, a 2016 study compared the efficacy of different washes and conditions (acid concentration, acid temperature, and water temperature) to reduce *Salmonella* contamination on the surface of pork and found that all of the treatments were equally effective [4]. Therefore, additional multi-hurdle approaches (use of multiple antimicrobial practices to achieve pathogen inhibition [5]) are needed to ensure the safety of pork meat.

Organic acids have been used as an effective antimicrobial against *Salmonella* in pork. Current literature suggests that lactic acid (LA), formic acid (FA), and peracetic acid (PA) can significantly reduce *Salmonella* counts on pork [1,6,7,8]. Food preservatives like potassium sorbate (PS) and sodium propionate (SOP) have also proved to reduce counts of Gram-negative bacteria [9,10]. All of these compounds have GRAS (Generally Recognized as Safe) status, making them suitable for use as antimicrobials in the meat industry. However, consumers are interested in the use of clean-label antimicrobial compounds, like essential oils (EOs). EOs are hydrophobic liquids mainly composed of aliphatic aldehydes, alcohols, and esters [11], and have previously showed promising antimicrobial effects against *Salmonella* in fruit, vegetable, and meat systems [12]. However, due to their dose- and matrix-dependent response, studies differ in the efficacy of EOs. For example, a recent 2020 study compared the effect of 0.2% and 0.4% oregano oil against *Salmonella* Typhimurium in broth [13]. Results showed 0.2% oregano oil did not significantly reduce bacterial counts, while 0.4% reduced *Salmonella* below the limit of detection. In the same study, oregano oil was used to inhibit *Salmonella* on chicken meat, where >0.8% oregano oil was necessary to significantly reduce *Salmonella* counts after 30 s at 4 °C. Furthermore, another study reported the effect of oregano oil against *Salmonella* on minced lamb and found that 0.6% oregano oil was enough to significantly reduce *Salmonella* counts [14]. Thus, these differences in effective levels in broth, chicken, and sheep meat exemplify the dose- and matrix-dependent response aspect of EOs.

To the best of our knowledge, no study has comprehensively screened the effect of multiple antimicrobials (like the ones mentioned), and their combinations, directly on pork to achieve a multi-hurdle approach. Response Surface Methodology (RSM) is a powerful and time- and cost-effective tool that can be used to comprehensively screen a high number of independent variables, such as antimicrobials, and their effect on a response, such as *Salmonella* cell counts, while performing a reduced number of experiments. Contrary to full factorial designs, RSM utilizes Design of Experiments (DOE) software to determine the least amount of runs needed to build a second-order regression model [15,16]. The objective of this study was to use RSM to study the effect of combinations of effective antimicrobial compounds to inhibit *Salmonella* Typhimurium cell counts on pork loin ends destined for comminuted products. The proposed screening method represents a real-life case scenario since (i) the tested substances were applied at regulatory acceptable levels, (ii) their effect was assessed against a food-borne relevant *Salmonella* Typhimurium strain, and (iii) all studies were performed directly on pork loin ends. 

## 2. Materials and Methods

Antimicrobial selection was carried out by identifying antimicrobials that are currently being used by the pork industry (FA, LA, and PA), chemical compounds that have shown antimicrobial activity in foods (PS and SOP), and chemical compounds that can potentially elicit inhibitory effects based on their chemical properties (EOs due to their phenolic compound content). Thirty-five compounds were selected to be screened for antimicrobial activity against *Salmonella* on pork.

### 2.1. Bacterial Strains and Inoculum Preparations

*Salmonella enterica* serovar Typhimurium (ATCC 19585) is a clinical isolate. The inoculum of *Salmonella* Typhimurium was prepared by inoculating Luria–Bertani (LB) broth (Fisher Scientific, Hampton, NH, USA) from glycerol stocks stored at −80 °C. The bacterium was grown overnight in 3 mL of LB broth for 18 h at 37 °C with shaking. The overnight culture was then serially diluted in phosphate-buffered saline (PBS) solution (137 mM NaCl, 2.7 mM KCl, and 10 mM phosphate buffer E404; VWR International, Radnor, PA, USA) to reach the desired inoculum. 

### 2.2. Antimicrobial Preparation of Organic Acids and Salts

The organic acids used in this study were lactic acid (VWR International, Radnor, PA, USA), peracetic acid (Sigma-Aldrich, St. Louis, MO, USA), and formic acid (Sigma-Aldrich, MA, USA). The salts used as antimicrobials were potassium sorbate (Sigma-Aldrich, MA, USA) and sodium propionate (Sigma-Aldrich, MA, USA). All solutions were prepared by diluting the compounds into distilled water to a final volume of 10 mL and were prepared fresh on the day of the experiment. See Table 1 for the final concentration of each compound on the pork disks.

### 2.3. Antimicrobial Preparation of EOs

An EO library composed of 30 oils (Sigma-Aldrich, Burlington, MA, USA) (Table 2) was tested for antimicrobial effects on the pork disk. The oils were emulsified to increase the affinity of the antimicrobial solution to the food matrix [17,18,19]. The emulsion was prepared as described before [17]. Briefly, the continuous phase of the emulsion was prepared with 3% Tween-80 (VWR International, Radnor, PA, USA) as a surfactant agent, 5% inulin from chicory (Sigma-Aldrich, MA, USA) as an emulsion stabilizer, and PBS. The EOs were added at a final concentration of 0.25–1% (based on the mass of the pork disk, Table 1), by preparing a stock solution of the antimicrobials, with a constant target volume of 400 μL. The solution was then covered with Parafilm (Bemis Company Inc., Terre Haute, IN, USA) to prevent volatilization during sonication. The EO solutions were sonicated (Branson 450 Digital Sonifier with probe, Marshall Scientific, Hampton, NH, USA) at 30% wave amplitude for 10 min in an ice bath. Emulsions were prepared fresh on the day of the experiment, and subsequently applied to the pork disk. 

### 2.4. Sample Preparation—Miniature Pork Disks

Pork loin ends in vacuum-sealed bags were obtained from Rantoul Foods in Rantoul, IL. After the initial 24 h holding period after slaughter, the loins were harvested by processor staff. The loin ends were separated from the loin manually and placed in a vacuum-sealed bag. Then, pork loin ends were collected by laboratory staff and immediately transferred to the laboratory (within 30 min) in an insulated bag to maintain the temperature of the meat below 10 °C. Upon receiving, the pork loin ends were refrigerated at 4 °C before further processing. Pork loin ends were sliced into 12 mm thick slices using a commercial deli meat slicer (Big Bite 8-1/2” meat slicer, LEM products, West Chester, OH, USA). The slices were then aseptically cut into cubes of 69 cm^3^ (0.55 ± 0.10 g approximately) by using a stainless steel blade with a grid of 7.6 by 7.6 mm (Zhejiang Fullstar Houseware Co., Taizhou, Zhejiang, China). Pork disks were then placed inside a 15 mL conical tube (VWR International, Radnor, PA, USA) for further experimentation. 

### 2.5. Initial Antimicrobial Screening

Each antimicrobial compound was tested individually on a pork disk to screen for single antimicrobial effects. Antimicrobial concentrations were calculated based on the weight of the pork disk. Once the pork disks had been placed inside conical tubes as described above, disks were inoculated by directly adding 150 μL of diluted *Salmonella* Typhimurium ATCC 19585 overnight culture on the surface of the meat to reach a final inoculum on the pork disk of approximately 4-log (CFU/g). The disks were incubated at 4 °C for 30 min to allow for cell attachment. Then, 400 μL of antimicrobial solutions was added onto the surface of the pork disk. Pork disks were incubated again at 4 °C for 30 min. Two controls were prepared: (i) a pork disk that was uninoculated to identify any *Salmonella* spp. contamination on the meat before treatment, and (ii) an inoculated disk not treated with antimicrobials to determine the inoculum concentration. Control disks were also incubated at 4 °C for 24 h. 

After 24 h, the pork disks were diluted 1:10 and 1:100 in PBS before spiral plating (Neutec group, Farmingdale, NY, USA) onto GranuCult Xylose Lysine Deoxycholate (XLD) agar (Sigma-Aldrich, Burlington, MA, USA) for selective *Salmonella* recovery. Plates were incubated at 37 °C for 24 h. Results are reported as the change in *Salmonella* cell counts after 24 h compared to the inoculum (cell counts at 0 h, Δ Cell counts).

Results were analyzed using GraphPad Prism 9 (GraphPad Software Inc., San Diego, CA, USA). A one-way ANOVA followed by Dunnett’s multiple comparisons test was performed to determine statistically significant differences (*p* < 0.05) between EOs and the untreated group. This experiment was performed in biological duplicate, that is, an independent set of pork disks for each replicate.

### 2.6. Design of Experiments to Build RSM Model

After initial screening, six of the eleven antimicrobial compounds that were most effective at reducing *Salmonella* Typhimurium counts were taken for further analysis. The selected antimicrobials were two organic acids that significantly inhibited *Salmonella* counts during the initial screening, and four of the nine EOs that showed inhibitory effects. Four EOs were chosen to represent the width of statistical significance found during the initial screening (*p* < 0.05–*p* < 0.0001).

A Doehlert matrix design was chosen as the DOE to build an RSM model (Doehlert matrix—RSM) due to its buildability characteristics [16,20,21,22,23,24]. A Doehlert uniform shell design for six factors was developed using MODDE 13 software (Sartorius AG, Gottingen, Lower Saxony, Germany). The software interface requires users to input independent variables and their range, as well as the model’s response. The model presented here included six independent variables (LA 0–1.25%, FA 0–0.25%, CLV 0–0.25%, CMN 0–0.25%, PPP 0–0.5%, and SPT 0–0.25%) and the selected response was expressed as the change in *Salmonella* cell counts after 24 h compared to the inoculum (Δ Cell counts) in log CFU/g. MODDE 13 automatically generates an experiment matrix that includes the necessary experiments to build a second-order linear model to explain the effect of single and paired interactions between the independent variables on the response. The experiment matrix (Table 3) included 42 experiments, 1 central point in triplicate (to calculate pure error), and 1 untreated control (inoculated yet non-treated disk) in duplicate.

As shown in Table 3, the experiment matrix requires the addition of all six antimicrobials to all 42 treatments. Considering that each antimicrobial was selected due to its individual inhibitory effect, antimicrobial concentrations had to be adjusted to prevent bacterial counts resulting from each treatment to be below the limit of detection (LOD). When the RSM models are fitted to a second-order linear model, actual bacterial counts need to be included. Counts below the LOD cause the model to reduce its prediction accuracy, since the data would not accurately reflect the effect of different antimicrobial doses on the response [24]. Therefore, the antimicrobial concentrations used for the Doehlert matrix—RSM were lower than those used during the initial screening (Table 1). 

Experiments were carried out following the experiment matrix in triplicate. However, each replicate was individually fitted to a second-order model to confirm that each replicate had the statistical quality parameters necessary to build an RSM model. The following parameters were considered to determine whether the obtained experimental data had enough quality to build the model: residual standard deviation (RSD), model regression *p*-value, lack of fit test, and linearity. Finally, the average of three replicates was used to generate the regression models that met quality parameters to reduce the RSD.

MODDE 13 software was used to analyze and fit the Doehlert matrix—RSM. The full quadratic model was reduced by deleting the quadratic effects of antimicrobials from the model since none of these showed to be significant (*p* > 0.1) to the model. 

### 2.7. Laboratory-Scale Validation of Antimicrobial Combination on Pork Slices

Pork loin ends were collected and sliced as mentioned in Section 2.3. The lab-scale validation protocol is based on the methodology previously described [7,25] with modifications. The laboratory-scale validation was carried out on pork slices with a surface area of approximately 50 cm^2^ (48.0 ± 3.0 g approximately). Slices were placed inside a Biosafety Cabinet (BSC) (1300 Series A2, Thermo Fisher Scientific, Waltham, MA, USA) lined with sanitized aluminum foil. Then, 200 μL of stationary phase *Salmonella* Typhimurium ATCC 19585 inoculum, diluted to 10^7^ CFU/mL (See Section 2.1), was added onto the slice to reach a desired inoculum concentration of approximately 4-log (CFU/g). Then, a sterile L-shaped rod (VWR International, Radnor, PA, USA) was used to distribute the inoculum over the surface of the slice. Slices were allowed to air-dry for 10 min before further treatment. Preliminary experiments showed that a 7-log inoculum resulted in a desired final concentration of 4-log CFU/g after spraying slices with 25 mL of DI water (non-treated control).

Sanitized Z-shaped metal hangers (Walmart Inc., Bentonville, AR, USA) were used to hang pork slices from a sanitized metal rod inside a BSC. An autoclave-safe bin was placed under the hanging slices to collect drippings from the meat. Antimicrobial treatments were added by using the same concentrations used in the original screening, also referred to as “working industry concentrations” (Table 1). 

A handheld gardening sprayer (Scotts Miracle-Gro Company, Marysville, OH, USA) was used to deliver 25 mL of antimicrobial solutions to the inoculated surface of the pork slice at a flow rate of approximately 4.1 mL/s. Pork slices were immediately transferred to an air-tight container (Sterilite Co., Townsend, MA, USA) where the slices hung from zip-ties during incubation at 4 °C for 24 h. Control slices used were non-inoculated (to identify any *Salmonella* spp. contamination on the meat before treatment) and untreated (inoculated slices used to determine the inoculum concentration).

For enumeration, slices were placed inside a Whirl-Pak bag (Whirl-Pak Filtration Group, Fort Atkinson, WI, USA) before adding enough PBS to reach an exact 1:5 dilution. Slices were then homogenized using a stomacher (Seward Stomacher 80 Biomaster Laboratory Blender, Seward Inc., Bohemia, NY, USA) at “normal” speed for 1 min. Slices were serially diluted to reach 1:10 and 1:100 dilutions with PBS and enumerated as described in 2.5. 

## 3. Results

### 3.1. High-Throughput Screening of Potential Antimicrobial Compounds Was Achieved Using a Miniaturized Pork Loin End Model

**Figure 1:**
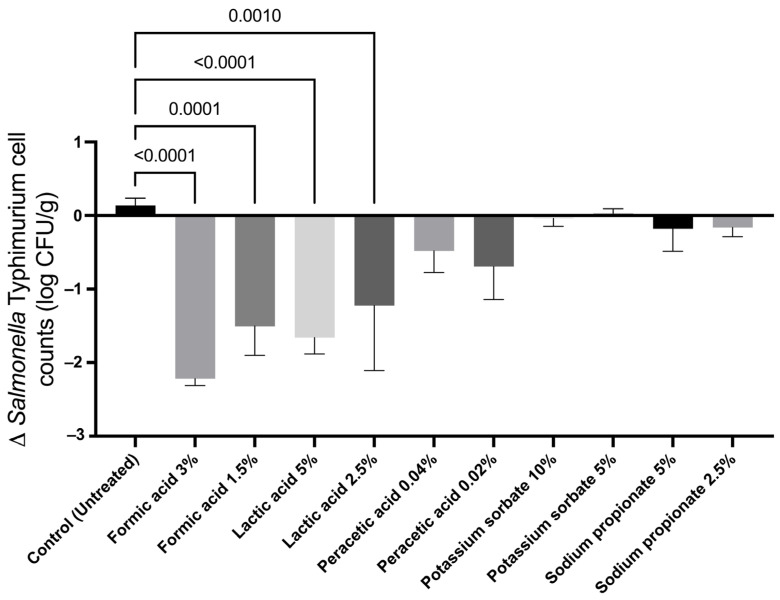
Antimicrobial screening of chemically polar compounds that were surface-applied to miniaturized pork disks previously inoculated with *Salmonella* Typhimurium ATCC 19585. Results are presented as the difference in cell counts after 24 h incubation at 4 °C; experiments were performed in biological triplicate. Antimicrobial treatments tested were formic acid (FA), lactic acid (LA), peracetic acid (PA), potassium sorbate (PS), and sodium propionate (SOP). Antimicrobial treatment concentrations are represented as % (*v*/*w*). Statistically significant treatments that had *p* < 0.05 from untreated sample have specific *p*-values annotated, while all other compounds had *p* > 0.05. Statistically significant compounds were identified through a one-way ANOVA followed by Dunnett’s multiple comparisons test.

A miniaturized pork model was developed by placing 69 cm^3^ cubes of pork loin ends into test tubes. This miniaturized food model allowed us to effectively screen many potential antimicrobial compounds directly on pork destined for comminuted products. Results of the initial screening of organic acids and common food preservatives at different levels (Table 1) can be found in Figure 1. LA was analyzed at 5% and 2.5% (*p* < 0.0001 and *p* = 0.001, respectively); results showed both concentrations to be statistically significant from the untreated control. Similarly, FA was analyzed at 3% and 1.5% (*p* < 0.0001 and *p* = 0.0002, respectively), where both concentrations were also found to be significantly different from the control group. Other screened compounds (PA, PS, and SOP) were not significantly different (*p* > 0.05) from the untreated control group, which might be due to the concentrations used in this study. These results confirm that LA and FA can be used as single antimicrobial treatments to reduce the *Salmonella* Typhimurium cell counts in pork.

**Figure 2:**
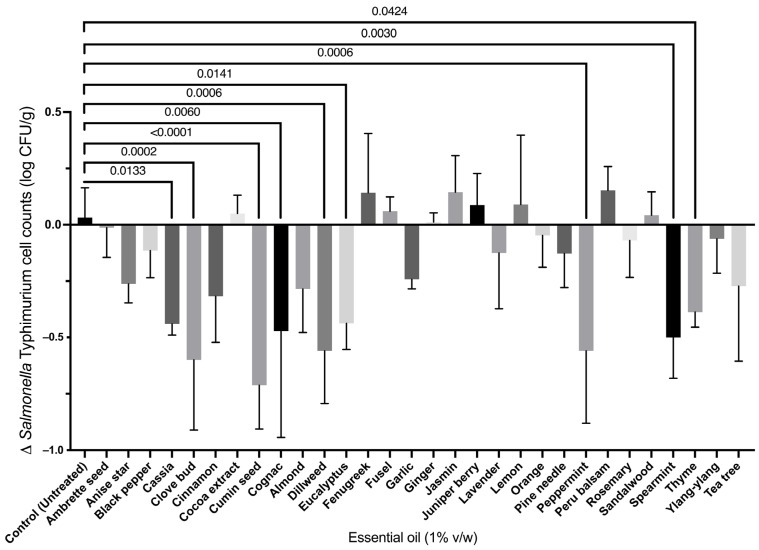
Antimicrobial screening of EO library. EOs were surface-applied to miniaturized pork disks previously inoculated with *Salmonella* Typhimurium ATCC 19585. Results are presented as the difference in cell counts after 24 h incubation at 4 °C. Experiments were carried out in biological duplicate. Statistically significant EOs were identified through a one-way ANOVA followed by Dunnett’s multiple comparisons test. Statistically significant treatments that had *p* < 0.05 from untreated sample have specific *p*-values annotated, while all other compounds had *p* > 0.05.

A library of 30 EOs (Table 2) was also screened for their potential use as antimicrobial treatments against *Salmonella* Typhimurium on miniaturized pork loin ends (Figure 2). The screening showed nine treatments of the 30 EOs used had significantly lower *Salmonella* counts than the untreated control group. CMN showed the highest antimicrobial activity against *Salmonella* Typhimurium (*p* < 0.0001), compared to the control group. CLV, dillweed, and PPP also showed significantly reduced *Salmonella* Typhimurium counts (*p* = 0.002, 0.006, and 0.006, respectively), followed by Cognac and SPT (*p* = 0.006 and 0.003, respectively), when compared to the untreated control group. Finally, cassia, eucalyptus, and thyme were also significant (*p* = 0.013, 0.014, and 0.042, respectively) when compared to the untreated control group for the reduction in *Salmonella* cell counts on a miniaturized pork disk.

Based on these results, six antimicrobial compounds were selected to be analyzed for possible interactions when applied in combination to pork. The selected antimicrobials represent the width of significance that was found during the initial screening. Nine EOs showed statistical significance that ranged from *p* < 0.05 to *p* < 0.0001. Therefore, six EOs were chosen to build a Doehlert matrix—RSM model, in addition to the two organic acids that showed significant inhibitory effects. The selected antimicrobial compounds were LA, FA, CMN, CLV, PPP, and SPT. 

### 3.2. A Doehlert Matrix—RSM Model Was Developed to Screen 2-Way Interactions between Antimicrobials and No Synergistic Interactions Were Identified

The Doehlert matrix was selected as the DOE for our analysis due to its buildability capacity. The Doehlert matrix relies on the development of an experiment matrix (Table 3) that contains the minimum number of experiments necessary to build a complete RSM linear regression model. However, The Doehlert matrix allows for the continuous addition of independent variables (antimicrobial compounds, in this case) even after the RSM model has been fitted [16,23]. This feature allows researchers to build into the already acquired knowledge from the first set of experiments by performing a few extra treatments without having to completely build a new experiment matrix. To the best of our knowledge, no other study has used Doehlert matrix—RSM to identify interactions solely between antimicrobial compounds to control bacterial growth on a food matrix.

The Doehlert matrix was built by using six independent variables (LA, FA, CMN, CLV, SPT, and PPP) at different concentrations (see Table 1), and one dependent variable, which was the change in *Salmonella* cell counts 24 h at 4 °C after treatment (represented as Δ *Salmonella* Typhimurium cell counts). As described in Section 2.6, the doses of antimicrobials were adjusted to build the Doehlert matrix—RSM model using actual bacterial counts (instead of <2.01 log CFU/g, LOD of spiral plating), which increases the accuracy of the model’s predictions [24]. For all antimicrobials, the lowest concentration used was 0%. 

The Doehlert matrix was used to gather experimental data that were then fitted into an RSM model. The obtained RSM linear regression model showed to be statistically significant (*p* < 0.0001), no lack of fit was detected (*p* = 0.248), and the RSD was 0.154 log CFU/g (Table 4). The obtained R^2^ value showed that 83% of the variance in the change in *Salmonella* cell counts can be explained by the dependent variables in the model (antimicrobial compounds). Therefore, the fitted Doehlert matrix—RSM model was appropriate for the analysis of antimicrobial interactions on the miniature pork loin end disks.

The experimentally obtained data for the Doehlert matrix were compared to the data predicted by the Doehlert matrix—RSM model (Table 3). The obtained residuals’ average was 6.7 × 10^−4^ log CFU/g, which suggests a strong correlation between the experimental and the predicted data. The Doehlert matrix—RSM linear regression model identified LA, CMN, PPP, and SPT as statistically significant (*p* = 0.0005, 0.003, 0.008, and 0.004, respectively) to the model when applied alone to the miniature pork disks (Table 5). Statistically significant terms in the model mean that varying the concentration of those antimicrobials has a significant effect on the change in *Salmonella* Typhimurium cell counts on pork. These results are to be expected, since the antimicrobials in the model (LA, FA, CMN, CLV, SPT, and PPP) showed significant effects against *Salmonella* in the initial screening results (*p* = 0.0010, 0.0001, <0.0001, 0.0002, 0.0030, and 0.0006, respectively) where they were tested individually (Figure 1 and Figure 2). Additionally, the model found CLV not to be significant to the model (*p* = 0.119). Furthermore, the 15 combinations that result from combining six antimicrobials in pairs showed no significant differences in the model (*p* > 0.1), thus suggesting that the Doehlert matrix—RSM model did not identify any two-way combination of antimicrobials to be significantly synergistic or antagonistic against *Salmonella* cell counts on pork meat. 

### 3.3. Lab-Scale Validation Confirmed Doehlert Matrix—RSM Model Results: Combining Organic Acids with EOs Reduces the Antimicrobial Activity of Organic Acids

A lab-scale validation was carried out to confirm the Doehlert matrix—RSM model predictions. The validation experiments included six antimicrobials tested alone, fifteen two-way combinations of these, and an untreated control group. Specific antimicrobial concentrations can be found in Table 1. The lab-scale validation was performed on pork loin slices that were left hanging for 24 h at 4 °C after the antimicrobial treatments were sprayed onto the surface of the meat. Results of the lab-scale validation (Table 5, column 5) showed that the untreated control group increased *Salmonella* Typhimurium cell counts by 0.09 log CFU/g. Results also showed that LA, FA, FA*CMN, FA*PPP, FA*CLV, and FA*SPT were significantly different from the untreated control group (*p* = 0.007, <0.001, 0.040, 0.042, 0.027, and 0.008, respectively). 

However, since the objective of the validation experiments was to confirm the results obtained by the Doehlert matrix—RSM model, the model predictions were compared to the results obtained by the validation experiments. Regardless of LA being significant (*p* = 0.007) when added alone to the pork slices, none of the two-way combinations of antimicrobials that include LA (LA*FA, LA*CMN, LA*PPP, LA*CLV, and LA*SPT) were significantly different from the control group (*p* > 0.05). Furthermore, LA*FA, LA*CMN, LA*PPP, LA*CLV, and LA*SPT are not significantly different from LA alone (*p* = 0.914, 0.847, 0.989, 0.706). Therefore, these results suggest that (i) adding LA alone is significantly better than no treatment, (ii) combining LA with any of the other tested antimicrobials is not significantly different from adding LA alone, and (iii) two-way combinations that include LA do not significantly change *Salmonella* cell counts compared to non-treated pork slices.

On the other hand, the combination of FA*SPT is not statistically different from adding FA alone (*p* = 0.099). Combinations of FA with CMN, PPP, and CLV were statistically different from adding FA alone (*p* = 0.022, 0.021, and 0.002, respectively) and from the untreated control (*p* = 0.040, 0.042, and 0.027, respectively). Additionally, the combination of FA with LA is not statistically different from the untreated control group (*p* = 0.303), thus suggesting that FA alone or FA combined with SPT are the most effective treatments for reducing *Salmonella* cell counts on pork slices, followed by FA*CMN, FA*PPP, and FA*CLV.

Finally, the lab-scale validation showed that CMN, PPP, CLV, and SPT alone were not significantly different from the untreated control group (*p* = 0.569, 0.801, 0.388, and 0.519, respectively). Similarly, two-way combinations between EOs (CMN*PPP, CMN*CLV, CMN*SPT, PPP*CLV, PPP*SPT, and CLV*SPT) did not show significant differences from the untreated control group (*p* > 0.05). Lab-scale results confirmed the previously obtained results from the Doehlert matrix—RSM model. Briefly, FA and LA are effective antimicrobials that can be used alone to reduce *Salmonella* Typhimurium cell counts on pork. Furthermore, no synergistic interactions were identified between the tested organic acids and EOs to control *Salmonella* cell counts.

## 4. Discussion

**Figure 3:**
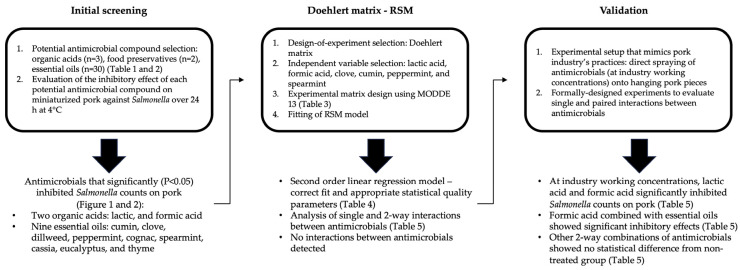
Experimental overview and specific results of the three major sections in the present study. Briefly, the results from the initial screening were used to design the Doehlert matrix—RSM. Similarly, the results from the Doehlert matrix—RSM were then used to set up the validation experiments.

Industry efforts to reduce *Salmonella* spp. cell counts on pork are mostly based on spraying organic acids directly onto the surface of the meat. However, due to lack of evidence of these kinds of treatments being significantly better than others (hot water sprays, dipping, higher acid concentrations, etc.) [4], concerns about negative impacts on product’s quality [9], and new consumer trends [26], an innovative approach to efficiently screen the antimicrobial potential of a large number of compounds was implemented and validated in this study. Our approach to screen a variety of chemically different compounds by implementing a miniaturized pork loin disk model allowed us to reduce the number of potential antimicrobial compounds from 35 to 11. A Doehlert matrix was used to develop an RSM model with the capacity to identify significant interactions between antimicrobials. The results showed no statistically significant interactions between tested antimicrobials added in pairs to pork meat to reduce *Salmonella* Typhimurium cell counts. Finally, a lab-scale validation that mimics the handling of pork cuts in a processing plant was implemented to validate the results from Doehlert matrix—RSM. Validation results confirmed the Doehlert matrix—RSM model’s predictions and provided insight into two-way combinations of the tested antimicrobial compounds. Figure 3 lays out the overall progression of experiments carried out in the present study, as well as specific results from each experimental stage.

### 4.1. High-Throughput Screening of EOs and Other Antimicrobials Found Most Do Not Show Antimicrobial Activity against Salmonella Typhimurium in Miniaturized Pork at Maximum Working Industry Concentrations

Considering the overwhelming number of potential antimicrobial compounds available, this study implemented a time-efficient method to evaluate antimicrobial activity against *Salmonella* Typhimurium in pork. The results obtained by the proposed screening method represent a real-life case scenario since (i) the tested substances were added at regulatory acceptable concentrations, (ii) their effect was assessed against a food-borne relevant *Salmonella* Typhimurium strain, and (iii) the studies were performed directly on the food matrix of interest, pork meat. The use of EOs as antimicrobials against *Salmonella* Typhimurium has been reported before in broth media. A 2015 study screened 21 EOs using the agar disk diffusion method against 10 strains of *Salmonella* sp. [27]. Results showed that CLV, cinnamon, oregano, thyme, bergamot, orange, cajeput, and sage oil were significantly effective against *Salmonella* spp. However, our results did not identify cinnamon, thyme, or orange to be effective using miniaturized pork loin disks. Another study in 2009 reported the effect of dipping pork loins into LA and acetic acid to reduce *Salmonella* sp. cell counts, as well as the effect of these acids in combination with supercritical carbon dioxide [28]. Results from the treatments with 3% LA showed a significant reduction in bacterial cell counts of 0.86 log CFU/cm^2^. However, LA treatments at 2.5% and 5% showed higher *Salmonella* Typhimurium cell reductions (1.51 and 1.66 log CFU/g, respectively) in the present study. These differences might be due to the considerably higher inoculum (7.02 log CFU/cm^2^) and to the inoculated samples being incubated overnight before antimicrobial treatments [28], which increases bacterial cell attachment, making antimicrobial treatments less effective. In 2022, the effect of adding LA and a lytic phage to a marinade (pH = 6.8) to reduce *Salmonella* sp. cell counts on pork loins was studied [29]. Results showed that 2.5% LA did not significantly inhibit *Salmonella* sp. compared to the inoculum (3.90 log CFU/cm^2^) after 1 h in the marinade. However, in the present study, 2.5% LA significantly reduced *Salmonella* counts by 1.2 log CFU/g (*p* = 0.001) compared to the control group. A possible reason for these two results to differ from each other might be that the higher pH and salt concentration of the marinade acts as a buffer, which impedes LA from dissociating in the matrix, making it less effective against bacteria [9]. These results emphasize the importance of screening antimicrobial treatments directly on the matrix of interest and designing experiments with the desired mode of application in mind. 

### 4.2. Formally Designed Experiments Can Be Used to Efficiently Screen Antimicrobial Treatments against Salmonella Typhimurium on Miniaturized Pork Disks

In the current study, we implemented an RSM model based on a Doehlert matrix (Doehlert matrix—RSM) to analyze the effect of six antimicrobials added alone or in pairs to control *Salmonella* Typhimurium cell counts on pork. RSM is an empirical modelling approach that is commonly used to optimize processes [16]. Central composite design (CCD) is the DOE most used for RSM models [30]; however, CCD is not the most efficient DOE. The Doehlert matrix uses the least number of experiments for the same number of variables, compared to CCD and Box–Behnken designs. Furthermore, the Doehlert matrix allows users to sequentially add new variables into the model by recycling the original DOE [23]. A 2001 study used a Doehlert matrix—RSM model to inhibit *Escherichia coli* through the combined effect of water activity (a_w_), pH, and nisin in peptone water [20]. Authors reported that all three of the selected factors significantly impacted *E. coli* cell counts. Regardless of the model showing a significant lack of fit, the linear regression model was used to optimize *E. coli* inhibition with good correlation (R^2^ = 0.947). Doehlert matrix—RSM has been extensively used in engineering, chemistry, and food analysis fields [22,31,32]. However, to the best of our knowledge, no other study has been published recently that implements a Doehlert matrix for food safety applications. 

Due to the nature of the Doehlert matrix, the independent variables in the model are applied together to every experiment in the matrix. Such an approach showed disadvantages in the current study, since the antimicrobial concentrations that showed inhibitory effects during initial screening were lowered significantly in the experiment matrix (Table 1). As expected, decreasing the concentration of the antimicrobials negatively affected their antimicrobial activity. For example, the Doehlert matrix—RSM predicted 0.25% FA to increase *Salmonella* cell counts (Table 5) regardless of screening results identifying 3% FA as the most efficient treatment (Figure 1). Furthermore, it is important to note that the Doehlert matrix—RSM developed in this study is a feasible tool to identify antimicrobial combinations that have a synergistic effect when combined against *Salmonella* Typhimurium. However, since the model requires all antimicrobials to be added at the same time onto the matrix, antimicrobial concentrations must be lowered significantly. Therefore, a secondary validation test must be implemented to find concentrations of synergistic antimicrobials that potentiate their effect on the food matrix, which is a potential disadvantage for the implementation of Doehlert matrix—RSM as a screening tool. However, a different DOE can be implemented to overcome the disadvantage of the Doehlert matrix, while still being more efficient than the traditional one-off experiment approach.

### 4.3. Pork Slices Were Used in a Lab-Scale Validation That Suggests EOs Decrease the Antimicrobial Activity of Tested Organic Acids against Salmonella Typhimurium

Results from Doehlert matrix—RSM were validated using a lab-scale validation. Current pork industry practices consist of spraying antimicrobials on the meat. Therefore, validation studies of antimicrobial efficacy should aim to mimic industry practices. A recent study [33] showed the effect of different meat surfaces on the antimicrobial activity of 3% LA and 0.04% PA. Pre-rigor skin-on pork carcasses were surface-inoculated with *Salmonella*, to a concentration of 5–6 log CFU/g. Antimicrobial treatments were applied by using an industrial sprayer. Results showed no significant differences in *Salmonella* counts between samples treated with LA or PA and the ambient temperature water control when applied to the skin-on surface of the carcass. However, LA and PA were significantly effective when applied to the inside of the body cavity of pork carcasses, which is mainly lean tissue. The results from the present study also showed that LA added alone to pork loins (mostly lean tissue) significantly inhibited *Salmonella* cell counts. However, it has been reported before that bacteria are more susceptible to organic acids when cells are attached to fatty tissue due to the reduced water activity. Less moisture reduces the chance of the acid being diluted or buffered by other components present on the surface of the matrix [9]. Therefore, antimicrobial interventions should be designed and selected considering the composition of the product that the treatment will be applied to. Furthermore, the current study showed no synergistic interactions between treatments, which is similar to what has been reported before. A study in 2019 reported the effect of six EOs, six organic acids, and their salts, alone and in combination, against *Salmonella* sp. in broth [34]. Results showed FA and CLV to be individually effective. However, two-way combinations between all tested compounds showed no synergistic effects; only additive effects were identified. Similarly, another study in 2005 combined five EOs and five organic acids against *Salmonella* Typhimurium in broth [35]. Results showed no synergistic effects between antimicrobials. However, the antimicrobial combinations were able to reduce bacteria cell counts. There is scarce information on the effect of combining antimicrobials directly on pork. However, this study provides insight into a possible antagonism from combining certain treatments in a lab-scale setting to inhibit *Salmonella* Typhimurium cell counts on pork. 

The current study presents an effective approach for antimicrobial screening on pork. Despite the limitations of the study, this is a viable option to study interactions between antimicrobials. The results from the present study also showcase that simply combining two effective antimicrobial compounds does not always lead to higher inhibition, which can potentially save money to industry when trying to implement novel antimicrobial interventions. Furthermore, the approach presented here represents a laboratory-scale validation that can easily be scaled up to industry, since the experimental design is based on current industry practices. Finally, the present study shows that antimicrobial screening should be designed and selected considering the composition of the product surface to which it is applied.

## 5. Conclusions

The current study implemented a high-throughput screening tool to screen new antimicrobial compounds as they become relevant for the pork industry directly on pork loin ends. Furthermore, once potential antimicrobials have been identified, a formally designed model can predict interactions between antimicrobials, or lack thereof. The model showed that LA, FA, CMN, PPP, and SPT can be used to efficiently inhibit *Salmonella* counts on pork. However, no two-way combinations between these antimicrobials showed significant antimicrobial inhibition. The predictions from the model were validated by spraying antimicrobials on pork loin end slices. The results obtained in this study showcase the importance of testing antimicrobials directly on the food matrix of interest, while keeping in mind the possible application of such interventions. This study exemplifies that simply combining antimicrobials that are individually effective does not necessarily lead to desired food safety outcomes. Product- and surface-specific antimicrobial treatments that prioritize practical implementation and ensure the desired quality parameters of the product are needed in the pork industry to reduce possible contamination of raw meat with foodborne bacteria. Further studies should focus on the implementation of RSM models for antimicrobial screening on food matrices that use other DOE to compare their efficacy to that of the Doehlert matrix.

## Figures and Tables

**Table 1:** Antimicrobial compounds tested on pork across different evaluations.

Antimicrobial	Code	Concentration (% *v*/*w*)		
		Initial Screening	Doehlert Matrix—RSM	Lab-Scale Validation
Formic acid	FA	1.5 and 3.0	0–1.25	3.0
Lactic Acid	LA	2.5 and 5.0	0–0.25	5.0
Peracetic acid	PA	0.02 and 0.04	NA	NA
Potassium sorbate	PS	5.0 and 10	NA	NA
Sodium propionate	SOP	2.5 and 5.0	NA	NA
Essential oils *	EOs	1.0	0–0.5	1.0

* Tested individually; see Table 2 for list of tested oils and abbreviations.

**Table 2:** Essential oil library screened for their individual antimicrobial activity against *Salmonella* Typhimurium ATCC 19585 on miniaturized pork disks.

#	Essential Oil	#	Essential Oil	#	Essential Oil
1	Almond	11	Dillweed	21	Orange
2	Ambrette	12	Eucalyptus	22	Peppermint (PPP) *
3	Anise star	13	Fenugreek	23	Peru
4	Black pepper	14	Fusel	24	Pine needle
5	Cassia	15	Garlic	25	Rosemary
6	Cinnamon	16	Ginger	26	Sandalwood
7	Clove (CLV) *	17	Jasmin	27	Spearmint (SPT) *
8	Cocoa extract	18	Juniper berry	28	Tea tree
9	Cognac	19	Lavender	29	Thyme
10	Cumin (CMN) *	20	Lemon	30	Ylang-ylang

* Oils that were selected to be used as independent variables in the Doehlert matrix—RSM. Food-grade oils, 99% purity.

**Table 3:** Designed Doehlert matrix for six antimicrobials (LA, FA, CLV, CMN, PPP, and SPT). Experimental data represent the mean of three biological replicates. Predicted data were calculated based on a linear regression model fitted to the experimental data.

Exp. No.	Antimicrobial Doehlert Matrix (%)						Experimental Data (log CFU/g)			Predicted Data (log CFU/g)	
	LA	FA	CMN	PPP	CLV	SPT	*Salmonella* Inoculum	*Salmonella* Cell Counts after 24 h	Δ *Salmonella* Cell Counts	Δ *Salmonella* Cell Counts	Residuals (Actual—Predicted)
1	1.25	0.13	0.13	0.25	0.13	0.25	4.05	3.1	−0.95	−0.88	−0.07
2	0.94	0.23	0.13	0.25	0.13	0.25	4.05	3.08	−0.97	−0.84	−0.13
3	0.94	0.16	0.23	0.25	0.13	0.25	4.05	3.07	−0.98	−1	0.02
4	0.94	0.16	0.15	0.45	0.13	0.25	4.05	3.15	−0.9	−0.96	0.06
5	0.94	0.16	0.15	0.29	0.22	0.25	4.05	3.12	−0.93	−1.05	0.12
6	0.94	0.16	0.15	0.29	0.14	0.44	4.05	3.21	−0.84	−0.94	0.1
7	0	0.13	0.13	0.25	0.13	0.25	4.05	3.38	−0.68	−0.42	−0.26
8	0.31	0.02	0.13	0.25	0.13	0.25	4.05	3.74	−0.31	−0.41	0.1
9	0.31	0.09	0.02	0.25	0.13	0.25	4.05	3.82	−0.23	−0.39	0.16
10	0.31	0.09	0.1	0.05	0.13	0.25	4.05	3.76	−0.29	−0.31	0.02
11	0.31	0.09	0.1	0.21	0.03	0.25	4.05	3.73	−0.32	−0.48	0.16
12	0.31	0.09	0.1	0.21	0.11	0.06	4.05	3.9	−0.15	−0.21	0.06
13	0.94	0.02	0.13	0.25	0.13	0.25	4.05	3.37	−0.68	−0.69	0.01
14	0.94	0.09	0.02	0.25	0.13	0.25	4.05	3.59	−0.46	−0.52	0.06
15	0.94	0.09	0.1	0.05	0.13	0.25	4.05	3.63	−0.43	−0.51	0.08
16	0.94	0.09	0.1	0.21	0.03	0.25	4.05	3.28	−0.77	−0.7	−0.07
17	0.94	0.09	0.1	0.21	0.11	0.06	4.05	3.56	−0.5	−0.48	−0.02
18	0.63	0.2	0.02	0.25	0.13	0.25	4.05	3.47	−0.58	−0.57	−0.01
19	0.63	0.2	0.1	0.05	0.13	0.25	4.05	3.72	−0.34	−0.48	0.14
20	0.63	0.2	0.1	0.21	0.03	0.25	4.05	3.57	−0.49	−0.52	0.03
21	0.63	0.2	0.1	0.21	0.11	0.06	4.05	3.62	−0.44	−0.51	0.07
22	0.63	0.13	0.2	0.05	0.13	0.25	4.05	3.23	−0.83	−0.76	−0.07
23	0.63	0.13	0.2	0.21	0.03	0.25	4.05	3.59	−0.46	−0.61	0.15
24	0.63	0.13	0.2	0.21	0.11	0.06	4.05	3.24	−0.82	−0.72	−0.1
25	0.63	0.13	0.13	0.41	0.03	0.25	4.05	3.57	−0.48	−0.58	0.1
26	0.63	0.13	0.13	0.41	0.11	0.06	4.05	3.58	−0.47	−0.52	0.05
27	0.63	0.13	0.13	0.25	0.21	0.06	4.05	3.37	−0.69	−0.61	−0.08
28	0.31	0.23	0.13	0.25	0.13	0.25	4.05	3.37	−0.69	−0.65	−0.04
29	0.31	0.16	0.23	0.25	0.13	0.25	4.05	3.47	−0.59	−0.66	0.07
30	0.31	0.16	0.15	0.45	0.13	0.25	4.05	3.29	−0.77	−0.68	−0.09
31	0.31	0.16	0.15	0.29	0.22	0.25	4.05	3.49	−0.57	−0.8	0.23
32	0.31	0.16	0.15	0.29	0.14	0.44	4.05	3.34	−0.71	−0.75	0.04
33	0.63	0.05	0.23	0.25	0.13	0.25	4.05	3.43	−0.62	−0.75	0.13
34	0.63	0.05	0.15	0.45	0.13	0.25	4.05	3.18	−0.88	−0.7	−0.18
35	0.63	0.05	0.15	0.29	0.22	0.25	4.05	3.47	−0.58	−0.67	0.09
36	0.63	0.05	0.15	0.29	0.14	0.44	4.05	3.14	−0.92	−0.82	−0.1
37	0.63	0.13	0.05	0.45	0.13	0.25	4.05	3.29	−0.77	−0.79	0.02
38	0.63	0.13	0.05	0.29	0.22	0.25	4.05	3.42	−0.63	−0.57	−0.06
39	0.63	0.13	0.05	0.29	0.14	0.44	4.05	3.24	−0.81	−0.85	0.04
40	0.63	0.13	0.13	0.09	0.22	0.25	4.05	3.5	−0.55	−0.52	−0.03
41	0.63	0.13	0.13	0.09	0.14	0.44	4.05	3.31	−0.75	−0.61	−0.14
42	0.63	0.13	0.13	0.25	0.04	0.44	4.05	3.24	−0.81	−0.78	−0.03
43	0.63	0.13	0.13	0.25	0.13	0.25	4.05	3.31	−0.74	−0.65	−0.09
44	0.63	0.13	0.13	0.25	0.13	0.25	4.05	3.24	−0.82	−0.65	−0.17
45	0.63	0.13	0.13	0.25	0.13	0.25	4.05	3.08	−0.98	−0.65	−0.33
46	0	0	0	0	0	0	4.05	4.23	0.18	0.23	−0.05
47	0	0	0	0	0	0	4.05	4.28	0.23	0.23	0

**Table 4:** Analysis of variance (ANOVA) for the fitted Doehlert matrix—RSM model. Doehlert matrix—RSM model consisted of six independent variables (LA 1.25%, FA 0.25%, CLV 0.25%, CMN 0.25%, PPP 0.5%, and SPT 0.5%) on the difference in *Salmonella* Typhimurium cell counts after 24 h at 4 °C.

Source	DF	Sum of Squares	Mean Square	F	*p*
Regression	21	2.91	0.14	5.80	<0.001
Residual	25	0.60	0.02		
Lack of fit	22	0.57	0.03	2.48	0.25
Pure error	3	0.03	0.01		
Total corrected	46	3.50	0.08		
R^2^	0.83	S.D.	0.28		
Adjusted R^2^	0.69	RSD	0.15		

**Table 5:** Doehlert matrix—RSM linear regression for all terms in the model and their corresponding coefficients and *p*-values (column 1–3). Fitted Doehlert matrix—RSM was used to predict *Salmonella* Typhimurium cell counts by using the model’s highest antimicrobial concentrations (column 4). Laboratory-scale validation results and statistical analysis for all terms in the final Doehlert matrix—RSM model (column 5).

Term	Coefficient (β) *	*p ^§^*	Predicted Δ *Salmonella* Cell Counts at Highest Doehlert Matrix—RSM Conc. **	Actual Δ *Salmonella* Cell Counts at Validation Conc. **^¶^
Constant	0.23	1.75 × 10^−20^	0.23	0.09 ^a^
LA	−0.32	0	−0.16	−1.23 ^bc^
FA	−0.44	0.07	0.12	−2.19 ^c^
CMN	−2.73	0.003	−0.45	−0.62 ^ab^
PPP	−0.12	0.008	0.17	−0.49 ^ab^
CLV	3.79	0.12	1.18	−0.56 ^ab^
SPT	−2.53	0.004	−1.03	−0.68 ^ab^
LA*FA	0.7	0.761	−0.27	−0.72 ^ab^
LA*CMN	−1.86	0.47	−0.85	−0.68 ^ab^
LA*PPP	−0.19	0.888	−0.22	−0.83 ^ab^
LA*CLV	0.11	0.968	0.78	−0.50 ^ab^
LA*SPT	0.48	0.719	−1.43	−0.61 ^ab^
FA*CMN	1.37	0.91	−0.56	−1.01 ^b^
FA*PPP	−1.29	0.841	0.06	−1.01 ^b^
FA*CLV	−15.28	0.251	1.07	−1.06 ^b^
FA*SPT	4.75	0.477	−1.14	−1.21 ^bc^
CMN*PPP	8.14	0.197	−0.51	−0.27 ^ab^
CMN*CLV	−14.61	0.261	0.5	−0.37 ^ab^
CMN*SPT	8.01	0.226	−1.72	−0.68 ^ab^
PPP*CLV	−6.47	0.304	1.12	−0.55 ^ab^
PPP*SPT	−1.94	0.545	−1.09	−0.61 ^ab^
CLV*SPT	2.98	0.631	−0.08	−0.41 ^ab^

* Coefficient values are unscaled and can be used to predict the change in *Salmonella* Typhimurium cell counts by multiplying them by any desired antimicrobial concentration within the limits of the model (see Table 1 and Table 2 for specific concentrations, and abbreviations). ^§^ Significant *p* < 0.1 represented in bold. ** Changes in *Salmonella* Typhimurium cell counts are represented as log CFU/g. ^¶^ Terms not connected by the same letter are statistically significant (α = 0.05) based on ANOVA analysis followed by a Tukey–Kramer multiple comparison test.

## Data Availability

The data used to support the findings of this study can be made available by the corresponding author upon request.
